# Production performance, egg quality and some blood parameters of heat‐stressed laying hens as affected by dietary supplemental Vit B6, Mg and Zn

**DOI:** 10.1002/vms3.737

**Published:** 2022-01-22

**Authors:** Hossein Gholizadeh, Mehran Torki, Hamed Mohammadi

**Affiliations:** ^1^ Animal Science Department College of Agriculture and Natural Resources Razi University Kermanshah Iran; ^2^ Department of Agriculture Payame Noor University Tehran Iran

**Keywords:** pyridoxine, zinc, magnesium, heat‐stressed laying hen

## Abstract

**Objectives:**

The effects of dietary supplements, including magnesium sulfate (Mg), zinc oxide (Zn) and vitamin B6 (Vit B6), on the performance of laying hens reared under normal (NC) and heat‐stress (HSC) conditions were investigated.

**Material and Methods:**

A total of 288 30‐week‐old Lohmann LSL‐Lite laying hens were randomly divided into 48 cages and assigned to receive one of the eight diets with six replicates and six hens per replicate, based on a 2 × 2 × 2 factorial arrangement of treatments. Dietary supplements, including two levels (0 & 600 mg/kg) of Mg, two levels (0 & 30 mg/kg) of Zn and two levels (0 & 8 mg/kg) of Vit B6, in normal and heat‐stress conditions, were tested at 30–40 and 41–45 weeks of age, respectively.

**Results:**

In the Vit B6 group, a decrease in feed intake (FI), egg production (EP), albumin, Zn, Fe and Mg, and an increase in triglyceride and insulin in HSC were observed, in addition to a decrease in cholesterol and an increase in egg weight (EW) in NC. Supplemental Mg decreased EP, blood triglycerides and copper in NC, and Zn, Fe and Mg in HSC as well. Feeding Zn, increased egg shape index, total protein and copper in addition to yolk index in NC and Fe in HSC. EWs were higher in hens supplemented with Vit B6+Mg in HSC. Increased insulin and decreased glutathione peroxidase activity were detected with the Vit B6+Zn compared to feeding either Vit B6 or Zn in HSC.

**Conclusions:**

The results indicated Vit B6 could improve EWs and suppress blood cholesterol in NC. Zn improved the egg shape index in NC.

## Graphical abstract

The results indicated that supplemental Vit B6, Mg and Zn did not improve the production performance of laying hens. Increased insulin and decreased glutathione peroxidase activity were detected with Vit B6+Zn compared to feeding either Vit B6 or Zn in HSC.

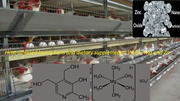

## 1 INTRODUCTION

There are several environmental factors that affect layer chickens in production, but the most debilitating is heat stress, which affects production parameters, especially for birds that are sensitive to heat waves because of their feather covering and lack of sweat glands, which makes heat dissipation difficult (Azzam et al., 2019; Estrada‐Pareja et al., 2007). Heat stress is a great concern in poultry industry because high ambient temperatures cause laying hens to lose body weight (Chand et al., 2016; Khan et al., 2014; Puthpongsiriporn et al., 2001; Mashaly et al., 2004) and negatively affect nutrient utilization, growth rate, EP and quality, feed efficiency and immunity; moreover, heat stress results in a higher incidence rate of oxidative stress in birds (Azzam et al., 2019; Khan et al., 2014). Heat stress may also increase mortality rate which leads to economic losses (Khan et al., 2011). Heat stress also decreases serum concentrations of vitamins, minerals and insulin and increases serum triglycerides, glucose, total cholesterol and corticosterone concentrations in poultry, therefore, heat stress may exacerbate a marginal vitamin and mineral deficiency or lead to increased mineral and vitamin requirements (Azzam et al., 2019; Khan et al., 2011). Various experiments have been conducted to alleviate negative consequences of high environmental temperatures (e.g., reducing dietary protein level, supplementing diet with minerals and vitamins) (Laudadio et al., 2012). Research on heat stress in laying hens indicates a consistent decrease in EW and shell thickness (Wolfenson et al., 2001). Synthesizing anti‐oxidant enzymes, such as superoxide dismutase (SOD) and glutathione peroxidase, is an important regulation, in terms of bird response to stress conditions. However, this response will be effective only if co‐factors, such as Se for glutathione peroxidase and Cu, Zn and Mn for SOD, are available (McDowell, 1989). Anti‐oxidant vitamins and minerals as a part of a nutritional manipulation tool are commonly added to the diets of birds reared under heat stress (Sahin & Kucuk, 2003a).

Zinc is used in poultry diets due to its anti‐stress effects. Moreover, its requirement increases and its retention decreases during stress (Sahin & Kucuk, 2003b). Copper‐zinc SOD (CuZnSOD) can catalyse the disproportionation of O2^−^ to H_2_O_2_ and oxygen. Zn is necessary for the structure and function of CuZnSOD; in addition, Zn is a main part of the alkaline phosphatase which plays an important role in calcium storage in bone (Li et al., 2019). Magnesium (Mg) is involved in many biochemical processes, including activation of phosphates and participation in carbohydrate metabolism, and its function is closely associated with calcium (Ca) and phosphorus (McDonald et al., 1971). Karami et al. (2018) stated that addition of single or combination of Cr, Zn and VitC improved at least some production performance and egg quality parameters in laying hens during heat‐stress condition; in addition, they detected an interaction effects of Cr, Zn and VitC on enzyme activity (glutathione peroxidase). The magnesium requirement of poultry has rarely been studied and most studies conducted are more than 35 years old (Shastak & Rodehutscord, 2015). Magnesium supplementation at high levels has been shown to improve EW (Atteh & Leeson, 1982); furthermore, high dietary Mg may influence Ca and P availability and metabolism (Shastak & Rodehutscord, 2015). Atteh and Leeson (1982) reported that Mg supplementation improves egg shell strength when Ca level is sufficient. Vit B6 is widely distributed in low concentrations in all animal and plant tissues (Atteh & Leeson, 1982). Pyridoxine (Vit B6) is an essential element for energy production, fat metabolism, central nervous system activity and haemoglobin production. In case of Vit B6 deficiency, slow growth and poor feed utilization in poultry has been observed. Stress increases Vit B6 requirement, so the practical recommendation is higher than the minimum requirement (Weber, 2009). On this basis, we hypothesized that inclusion of Vit B6, Mg and/or Zn into laying hens' diets would be helpful to enhance production performance and have antioxidant benefits in heat‐stress condition. The aim of the present study was to investigate this hypothesis in heat‐stressed laying hens (30–40 and 41–45 weeks of age) whose diets have been supplemented with Vit B6, Mg and/or Zn.

## 2 MATERIALS AND METHODS

### 2.1 Animals, treatments, and management

A total number of 288 30‐week‐old Lohmann LSL‐Lite laying hens were randomly divided into 48 cages and assigned to receive one of the eight experimental diets of six replicates and six hens per replicate. Based on a 2 × 2 × 2 factorial arrangement of treatments, eight diets consisted of two levels (0 and 600 mg/kg) of Mg sulfate, two levels (0 and 30 mg/kg) of zinc oxide, and two levels (0 and 8 mg/kg) of Vit B6 were formulated. The selected dietary supplemental pyridoxine levels were based on Karami et al. (2018), Sahin et al. (2002), Sedgh‐Gooya and Torki (2018) and Kucuk et al. (2008). The ingredients and chemical composition of the basal diet (control) (NRC, 1994) is shown in Table 1. All hens were supplied with feed and water ad libitum in the experimental period. The ambient temperature of the hens was kept from 18 to 20°C during the first 10‐week (30 to 40 weeks of age) and then (41 to 45 weeks of age) was gradually increased to 30 ± 1°C to simulate thermal heating on a hot day (Azzam et al., 2019; Xing et al., 2019). The relative humidity was kept as close as possible to a constant 40 to 60%, and hen house was lit for 16 h/day during the experimental period.

**TABLE 1:** Ingredients and nutrient composition of the basal diet (%, unless stated otherwise)

Ingredients	
Corn	65.12
Soybean meal	22.99
Soybean oil	0.69
Oyster shell	8.78
Dicalcium phosphate	1.39
Common salt	0.34
Vitamin premix^a^	0.25
Mineral premix^b^	0.25
DL‐methionine	0.21

Nutrient composition	
Metabolisable energy (kcal/kg)	2720
Crude protein (%)	16.82
Lysine (%)	0.59
Methionine + Cysteine (%)	0.46
Calcium (%)	3.73
Non‐phytate phosphorus (%)	0.38
Chromium (mg/kg)	5.43
Zinc (mg/kg)	84.00

^a^ Vitamin mixture per 2. 5 kg of diet provides the following: vitamin A, 7,700,000 IU; vitamin D_3_, 3,300,000 IU; vitamin E, 6600 mg; vitamin K_3_, 550 mg; thiamine, 2200 mg; riboflavin, 4400 mg; vitamin B6, 4400 mg; capantothenate, 550 mg; nicotinic acid, 200 mg; folic acid, 110 mg; choline chloride, 275,000 mg; biotin, 55mg; vitamin B_12_, 8. 8 mg

^b^ Mineral mixture per 2.5 kg of diet provides the following: Mn, 66,000 mg; Zn, 66,000 mg; Fe, 33,000 mg; Cu, 8800 mg; Se, 300 mg; I, 900 mg

### 2.2 Production performance

Production performance of the laying hens was measured from 30 to 45 weeks of age. Daily EP per replicate was recorded, and the total number of eggs laid per bird was calculated before and after heat treatment. Similarly, the eggs laid per replicate were weighed daily, and the average egg mass per bird was calculated before and after heat treatment. FI values were measured on a weekly basis. Egg mass (g egg/hen/day) and feed conversion ratio (FCR) (g feed/g egg) were calculated from EP, egg mass and FI.

### 2.3 Egg quality

Random samples of 12 eggs from each treatment (3 eggs per replicate) were collected at 36 and 44 weeks of age to measure egg quality traits. The eggs were broken on a glass plate to measure the albumen height using a micrometre, and Haugh units were calculated using the formula described by Eisen et al. (1962). The egg‐specific gravity was determined using 11 gradient saline solutions varying in specific gravity from 1.060 to 1.100 at 0.005‐unit increments (Holder & Bradford, 1979). The eggshell thickness were determined by taking the mean value of the thickness measured at three locations on the egg (air cell, equator and sharp end) using an FHK device (Fujihira Industry Co. Ltd., Saitama, Japan). The yolk index was determined as the ratio of yolk height to yolk width and yolk colour was compared to the Roche yolk color fan, which ranges from pale yellow at score 1 to dark orange at score 15 (Vuilleumier, 1969).

### 2.4 Blood parameters

Blood samples were obtained from the wing vein of four randomly selected birds per treatment (one hen per replicate) at 40 and 45 weeks of age. Blood samples were collected in heparinised and non‐heparinised vacutainer tubes. The collected blood samples were centrifuged at 3000 rpms for 10 min to collect plasma and serum and were then frozen at 20°C until the analysis. The serum concentrations of total protein, albumin, glucose, TG, total cholesterol and insulin were measured using commercially available kits (the catalogue numbers and company names are: 128500 [Pars Azmoon, Tehran, Iran], 101500 [Pars Azmoon], 117500 [Pars Azmoon], 132500 [Pars Azmoon], 110500 [Pars Azmoon] and 8K41‐25‐16221LP49 [Abbott Diagnostics], respectively), according to colorimetric enzymatic method, and the serum glutathione peroxidase activity was measured according to Paglia and Valentine (1967). The serum concentrations of Mg, Zn, Fe and Cu were determined using commercially available kits (the catalogue numbers and company names are: 126500 [Pars Azmoon], L279/12 [Ziest Chemie Co., Iran] and 121400 [Pars Azmoon] respectively), via an atomic absorption spectrophotometer (Perkin Elmer HGA 500) with a graphite furnace atomizer in deuterium background correction method.

### 2.5 Statistical analysis

The data were subjected to ANOVA in a completely randomized design with a 2 × 2 × 2 factorial arrangement of treatments using GLM procedure of SAS software (SAS 2003). All statements of significance are based on *p* < 0.05. The mean values were compared by Duncan's multiple‐range tests. The following model was considered for analysis: *Y*_ijkl_ = *μ* + (*A*_i_) + (*B*_j_) + (*C_k_*) + (AB_ij_) + (AC_ik_) + (BC_jk_) + (ABC_ijk_) + (e_ijkl_), where *Y*_ijkl_ is the measured characteristic, *μ* is the overall mean, (*A_i_*) is the main effect of Vit B6, (*B_j_*) is the main effect of Mg, *C_k_* is the main effect of Zn, AB_ij_ is interaction between the Vit B6 and Mg, AC_ik_ is interaction between the Vit B6 and Zn, BC_jk_ is interaction between the Mg and Zn, ABC_ijk_ is interaction between the Vit B6, Mg and Zn, and *e_ijkl_* is the residual error. The effects of the main factors were not considered whenever the interaction was significant.

## 3 RESULTS AND DISCUSSION

### 3.1 Production performance

The effects of dietary supplemental Zn, Mg and Vit B6 on the performance of laying hens are shown in Table 2. High ambient temperature is considered to influence the production performance in poultry adversely. Some studies showed that heat stress reduces FI, EP, EW and increases FCR (Mashaly et al., 2004; Puthpongsiriporn et al., 2001). Corticosterone and catecholamine production and cell membrane lipid peroxidation may be increased in heat‐stress conditions (Puthpongsiriporn et al., 2001). It is believed that for every 10°C increase in ambient temperature above 20°C, there is a 17% reduction in FI, suggesting that reduction in efficiency could be due to changes in metabolic utilization of nutrients (Geraert et al., 1996). In this study, the oral administration of Zn, Mg and Vit B6 in the diet did not influence FI preceding heat‐stress exposure. However, under heat stress, Vit B6 reduced FI, but the other supplements did not have any effects. Pyridoxine influences the control of food intake through their roles in neurotransmitter synthesis and regulation in the central nervous system, since it is involved in the hydroxylation and decarboxylation of tryptophan for the synthesis of serotonin (Groff & Gropper, 2000), which is known to control food intake through the melanocortin pathways and helps to control appetite and ward off eating (Heisler et al., 2003). Karami et al. (2018) stated that addition of single or combination of Cr (400 μg/kg diet), Zn (30 mg/kg diet) and VitC (250 mg/kg diet) improved at least some production performance parameters in laying hens during heat‐stress condition. Low Vit B6 status and its resulting decrease in brain serotonin synthesis (Anderson & Johnston, 1983) may result in an increased appetite and presumably food intake and favour a positive energy imbalance in individuals.

**TABLE 2:** Mean comparison effects of Vit B6, Mg and Zn on production performance of laying hens reared under normal (weeks 30 to 40 of age) and heat‐stress (weeks 41 to 45 of age) conditions

	Egg production (%)			Egg mass		Egg weight (g)			Feed intake (g)		Feed conversion ratio	
Age (weeks)	30–35	35–40	40‐45	30—40	40–45	30‐35	35‐40	40‐45	30‐40	40‐45	30‐40	40‐45
Condition	NC^a^	NC	HSC^b^	NC	HSC	NC	NC	HSC	NC	HSC	NC	HSC
Treatments												
Vit B6												
0 mg	96.84±3.30	95.75±3.64	94.66±3.38^*^	56.65±1.70	55.37±2.08	57.91±1.36	59.08±1.02^b^	58.48±1.34	109.91±0.19	109.74±0.54^*^	1.59±0.08	1.96±0.07
8 mg	95.21±5.87	94.15±2.27	91.68±5.23^b^	57.13±2.48	53.99±3.08	58.67±1.61	59.82±1.40^a^	58.91±1.50	109.71±0.45	109.71±0.61^b^	1.98±0.15	1.99±0.11
MgSo4												
0 mg	97.59±2.25^*^	96.55±1.46^a^	93.71±3.58	57.41±1.24	54.89±2.46	58.31±1.36	59.45±1.24	58.57±1.31	109.83±0.31	109.97±0.09	1.94±0.11	1.97±0.09
600 mg	94.56±5.98^b^	93.45±5.85^b^	92.64±5.47	56.32±2.63	54.47±2.95	58.27±1.27	59.45±1.33	58.81±1.55	109.79±0.50	109.94±0.18	1.99±0.13	1.98±0.10
ZnO												
0 mg	96.20±4.83	95.18±4.36	92.83±4.82	56.97±2.27	54.47±2.87	58.47±1.54	59.52±1.39	58.68±1.69	109.80±0.49	107.74±2.21	1.96±0.12	1.98±0.08
30 mg	95.88±4.78	94.76±4.78	93.52±4.46	56.78±1.94	54.89±2.45	58.11±1.52	59.38±1.16	58.71±1.13	109.82±0.32	108.39±2.07	1.97±0.12	1.97±0.09
SEM*	0.694	0.662	0.665	0.319	0.389	0.22	0.18	0.20	0.059	0.309	6.34	4.95
CV	4.95	4.78	4.95	3.68	4.93	2.62	2.14	2.43	0.37	1.98	0.018	0.014

Source of variations						P‐value						
Vit B6	0.27	0.24	0.02	0.44	0.09	0.09	0.04	0.28	0.10	0.05	0.51	0.28
MgSo4	0.03	0.01	0.41	0.09	0.60	0.92	0.98	0.54	0.69	0.54	0.22	0.74
ZnO	0.87	0.70	0.59	0.77	0.59	0.43	0.69	0.93	0.88	0.28	0.66	0.87
Vit B6 × MgSo4	0.30	0.54	0.85	0.66	0.72	0.52	0.55	0.22	0.52	0.25	0.89	0.40
Vit B6 × ZnO	0.30	0.37	0.29	0.60	0.57	0.99	0.66	0.30	0.82	0.55	0.51	0.64
MgSo4 × ZnO	0.59	0.86	0.94	0.99	0.75	0.96	0.53	0.61	0.54	0.67	0.72	0.52
Three‐way interaction	0.87	0.16	0.12	0.99	0.64	0.48	0.17	0.02	0.09	0.24	0.82	0.39

Values in the same column with different superscripts are significantly different (*p* < 0.05).

* SEM, standard error of the mean.

^a^ Normal condition.

^b^ Heat‐stress condition.

The reduction in FI in this study could be related to heat stress and feeding excess Vit B6 (Nachtigal et al., 2005). In agreement with this study, previous researchers reported that the level of Zn in the diet did not significantly influence broiler performance, FI and FCR (Hamidi et al., 2010). In this research, the combination of Vit B6 and Zn did not influence FI and FCR. In contrast, in a more comprehensive experiment, Kucuk et al. (2008) reported that FCR, EP, eggshell weights and Haugh units were improved when both Zn and Vit B6 were supplemented to laying hens.

In the present study, no effects of dietary Vit B6 were found on EP before heat‐stress exposure (30 to 35, and 35 to 40 weeks of age), while under heat stress (40 to 45 weeks of age), there was a decrease in EP in receiving Vit B6 group. The decrease in EP could be related to the numerical decrease in FI in receiving Vit B6 group under heat stress. In this experiment, feeding Zn did not have any effects on EP either before or during heat‐stress exposure. In contrast, Sahin & Kucuk (2003) reported linear increases in FI, EP, egg quality and improved feed efficiency upon ZnSO_4_ supplementation (30 and 60 mg/kg of diet) to quail reared under heat‐ stress conditions. In a study, ileal villus height was increased (*p* < 0.05) with supplementation of 60 mg/kg Zn compared with control group (Shah, et al., 2018).

Feeding Mg caused a decrease in EP before heat‐stress exposure (30 to 35, and 35 to 40 weeks of age), but did not cause any effect during heat‐stress exposure. Stafford and Edwards (1973) suggested that as long as the level of Mg content has reached the minimal requirement, an excessive supplementation of Mg in the diets would result in neither positive nor negative effects to EP of laying hens. In this research, increasing dietary Mg did not influence FI. Liu et al. (2007) reported that increasing Mg linearly reduced FI. In this experiment, the hens that were fed excess Mg before heat‐stress exposure, ate less compared to those who were under heat stress. The differences in feed consumption were non‐significant, but the numerical values were lower for the hens before heat‐ stress exposure. Therefore, the reduction in feed consumption caused by excess Mg might affect EP, and the decline in EP could have been related to decreased feed consumption. It also appears that the hens were fed high Mg‐adjusted feed consumption to allow intake to be near normal.

The increased levels of Mg in the blood and bones of the hens fed excess Mg suggest a possible mechanism for the action of the Mg. It has been shown that blood Mg level plays an important role in the control of parathyroid hormone release and that Mg may be as effective as Ca on a molar basis in parathyroid gland function (Sherwood et al., 1970). Parathyroid hormone release is reduced by both high and low levels of dietary Mg (MacManus et al., 1971). Mg excess has been shown to suppress parathyroid activity in dogs (Massry et al., 1970) and rats (Clark, 1969). In broilers, excess Mg altered the histological appearance of the parathyroid gland (Lee & Britton, 1980). It is suggested that the mechanism by which excess Mg has its effect in layers is to reduce parathyroid hormone activity, which reduces blood Ca, and thus, shell quality and EP are affected. In this experiment, there was no effect of feeding Zn on EP. Several studies also reported no effect of Zn addition on EP (Kidd et al., 1992). However, Paulicks and Kirchgessner (1994) found a positive effect of Zn supplementation on EP in lying hens; however, this result could be due to the concentration of Zn in the diet, 20 mg/kg, which was quite lower than the recommended concentration (50 mg/kg) (Underwood & Suttle, 1999). Since a wide variety of variables, such as supplementation methods (in diet or drinking water), the supplemental level, its level in the basal diet, bioavailability, stress condition, degree of stress, as well as duration of usage may influence the results, they could be, at least in part, probable reasons for various results in miscellaneous experiments.

### 3.2 Egg quality

Interaction effects between the Vit B6 (a), Mg (b) and Zinc (c) on egg weight, shell weight, shell thickness values and insulin, Zn and Mg levels (Table 4, 4) were significantly different in heat‐stress (weeks 41 to 45 of age) condition (Table 5); besides, in normal condition (weeks 30 to 40 of age), interaction effects between the supplements on Haugh unit values were significant (Table 5). In this research, Zn supplementation did not affect the EW of the birds consuming Zn compared to the others (Table 3). In agreement, in a study by Kidd et al. (1992) with broiler breeders, no effect of increasing Zn concentration on EW was found. They reported that eggshell weights and Haugh units were also increased when both Vit B6 and Zn were supplemented, but in our study, feeding both Vit B6 and Zn had no influence on Haugh unit and eggshell weights. Karami et al. (2018) stated that the addition of a single or combination of Cr, Zn and VitC (400 μg/kg diet, 30 mg/kg diet and 250 mg/kg diet, respectively) improved some egg quality parameters in laying hens during heat‐stress conditions. Dietary supplementation of 80 (Moreng et al., 1992) or 100 mg/kg of Zn (Balnave & Zhang, 1993) as Zn‐methionine was shown to improve eggshell weight and reduce shell defects in hens exposed to high temperatures. Since Zn is a constituent of carbonic anhydrase enzyme, it may affect eggshell properties (Nys et al., 2001).

**TABLE 3:** Mean comparison effects of Vit B6, Mg and Zn on egg quality parameters of laying hens reared under normal (weeks 30 to 40 of age) and heat‐stress (weeks 41 to 45 of age) conditions

	Egg shape index (%)		Yolk color		Yolk index (%)		Haugh unit		Shell weight (g)		Shell thickness (mm^2−^10 ×)	
Age (week)	30–40	40–45	30–40	40–45	30–40	40–45	30–40	40–45	30‐40	40–45	30‐40	40–45
Condition	NC^a^	HSC^b^	NC	HSC	NC	HSC	NC	HSC	NC	HSC	NC	HSC
Treatments												
Vit B6												
0 mg	74.46±1.23	73.21±2.34	6.83±0.49	5.98±0.27	41.2±1.56	37.71±1.14	69.58±3.15	73.17±3.44	5.96±0.19	5.49±0.26	0.37±0.008	0.34±0.01
8 mg	74.16±1.18	73.41±1.23	6.81±0.56	5.91±0.34	40.99±1.76	37.40±1.14	70.87±3.05	74.17±3.00	6.07±0.19	5.40±0.33	0.38±0.01	0.33±0.01
MgSo4												
0 mg	74.24±1.27	73.18±1.29	7.07±0.51^a^	5.88±0.31	40.94±1.85	37.51±1.27	69.98±3.51	72.98±3.83	6.00±0.20	5.43±0.33	0.37±0.01	0.33±0.01
600 mg	74.39±1.15	73.45±2.31	6.58±0.40^b^	6.01±0.30	41.07±1.44	37.60±1.00	70.47±2.77	74.36±2.40	6.02±0.19	5.46±0.27	0.38±0.009	0.34±0.01
ZnO												
0 mg	73.93±1.13^b^	72.81±1.27	6.87±0.48	5.99±0.26	41.49±1.45	37.66±1.31	70.00±3.18	72.96±3.47	5.99±0.21	5.49±0.25	0.38±0.01	0.34±0.01
30 mg	74.67±1.17^a^	73.82±2.21	6.77±0.56	5.89±0.35	40.52±1.71	37.45±0.95	70.45±3.15	74.38±2.88	6.03±0.18	5.39±0.33	0.37±0.008	0.34±0.01
SEM*	0.175	0.268	0.075	0.046	0.237	0.164	0.453	0.467	0.02	0.043	0.001	0.002
CV	1.61	2.53	7.67	5.25	4.02	4.03	4.47	4.39	3.36	5.56	2.59	4.96

Source of variations						P‐value						
Vit B6	0.39	0.71	0.91	0.47	0.94	0.33	0.13	0.26	0.07	0.30	0.30	0.57
MgSo4	0.65	0.61	0.01	0.20	0.77	0.76	0.56	0.13	0.66	0.71	0.48	0.26
ZnO	0.04	0.06	0.50	0.29	0.03	0.52	0.59	0.12	0.57	0.25	0.30	0.34
Vit B6 × MgSo4	0.86	0.60	0.92	0.29	0.26	0.63	0.01	0.94	0.15	0.47	0.64	0.85
Vit B6 × ZnO	0.44	0.18	0.92	0.89	0.87	0.18	0.74	0.64	0.50	0.33	0.07	0.70
MgSo4 × ZnO	0.96	0.19	0.77	0.47	0.05	0.02	0.79	0.14	0.36	0.39	0.90	0.34
Three‐way interaction	0.26	0.88	0.92	0.28	0.27	0.53	0.04	0.09	0.19	0.03	0.30	0.01

Values in the same column with different superscripts are significantly different (*p* < 0.05).

* SEM, standard error of the mean.

^a^ Normal condition.

^b^ Heat‐stress condition.

**TABLE 4:** Mean comparison effects of Vit B6, Mg and Zn on blood parameters of laying hens reared under normal (weeks 30 to 40 of age) and heat‐stress (weeks 41 to 45 of age) conditions

	Triglycerides (mg/dL)		Cholesterol (mg/dL)		Total protein (g/dL)		Albumin (g/dL)		Glucose (mg/dL)	
Age (week)	30‐40	40–45	30–40	40–45	30–40	40–45	30–40	40–45	30–40	40–45
Condition	NC^a^	HSC^b^	NC	HSC	NC	HSC	NC	HSC	NC	HSC
Treatments										
Vit B6										
0 mg	1791.44±389.79	1787.43±536.93	269.32±87.02^a^	203.72±50.11	5.38±1.02	5.68±0.99	3.16±0.30	3.56±0.71^a^	369.23±144.73	499.03±198.73
8 mg	189.61 ± 66.06	2129.06 ± 641.25	221.80 ± 70.77^b^	200.81 ± 32.67	5.38 ± 0.78	5.56 ± 0.86	3.16 ± 0.52	3.10 ± 0.50^b^	302.53 ± 105.73	541.95 ± 189.09
MgSo4										
0 mg	1913.74 ± 484.06	1801.03 ± 685.45	254.49 ± 93.44	207.92 ± 36.22	5.33 ± 0.90	5.62 ± 1.09	3.17 ± 0.42	3.39 ± 0.72	428.19 ± 139.22	539.93 ± 231.52
600 mg	1776.56 ± 332.82	2077.51 ± 485.53	236.63 ± 69.73	196.90 ± 47.17	5.43 ± 0.91	5.63 ± 0.74	3.15 ± 0.45	3.28 ± 0.59	262.43 ± 53.89	500.78 ± 154.89
ZnO										
0 mg	1797.65 ± 419.93	1957.12 ± 682.81	230.86 ± 69.08	209.77 ± 38.57	5.12 ± 0.83	5.88 ± 0.94	3.18 ± 0.33	3.25 ± 0.48	360.76 ± 166.56	443.27 ± 175.93
30 mg	194.4.38 ± 419.52	1921.42 ± 528.35	260.26 ± 92.39	195.13 ± 44.77	5.68 ± 0.90	5.36 ± 0.84	3.14 ± 0.51	3.42 ± 0.79	315.18 ± 76.39	590.18 ± 183.40
SEM*	66.88	100.32	11.84	6.13	0.140	0.138	0.064	0.096	21.53	30.08
CV	22.61	31.04	33.41	20.78	16.73	16.47	13.67	19.70	38.68	37.12

Source of variations					P‐value					
Vit B6	0.47	0.05	0.01	0.84	0.71	0.63	0.89	0.01	0.01	0.19
MgSo4	0.05	0.26	0.34	0.38	0.59	0.95	0.74	0.66	0.01	0.60
ZnO	0.24	0.32	0.12	0.25	0.03	0.07	0.66	0.31	0.01	0.01
Vit B6 × MgSo4	0.01	0.48	0.97	0.58	0.98	0.94	0.13	0.59	0.48	0.08
Vit B6 × ZnO	0.01	0.01	0.26	0.34	0.01	0.20	0.48	0.09	0.01	0.27
MgSo4 × ZnO	0.01	0.01	0.01	0.83	0.27	0.30	0.84	0.49	0.01	0.04
Three‐way interaction	0.57	0.12	0.07	0.30	0.52	0.23	0.85	0.96	0.14	0.27

Values in the same column with different superscripts are significantly different (*p* < 0.05).

* SEM, standard error of the mean.

^a^ Normal condition.

^b^ Heat‐stress condition.

**TABLE 4:** (continued) Mean comparison effects of Vit B6, Mg and Zn on blood parameters of laying hens reared under heat‐stress (weeks 41 to 45 of age) conditions

	Glutathione peroxidase activity	Insulin μ (IU/mL)	Zinc μ (g/mL)	Fe μ (g/mL)	Heterophil:lymphocyte ratio	Mg μ (g/mL)	Cu μ (gg/mL)
Age (week)	45	45	45	45	45	45	45
Condition	HSC^a^	HSC	HSC	HSC	HSC	HSC	HSC
Treatments							
Vit B6							
0 mg	645.12 ± 274.75	26.23 ± 18.55	13.67 ± 3.46	272.24 ± 181.91	0.40 ± 0.21	1.60 ± 0.21	1.25 ± 0.81
8 mg	611.34 ± 307.56	53.06 ± 35.94	11.43 ± 3.14	61.70 ± 33.62	0.34 ± 0.14	1.45 ± 0.22	1.26 ± 0.68
MgSO_4_							
0 mg	571.01 ± 190.85	39.22 ± 37.25	13.91 ± 2.43	286.09 ± 196.95	0.42 ± 0.16	1.60 ± 0.18	1.05 ± 0.59
600 mg	691.15 ± 337.87	36.43 ± 23.30	11.19 ± 3.83	107.69 ± 108.88	0.31 ± 0.19	1.46 ± 0.25	1.48 ± 0.83
ZnO							
0 mg	691.27 ± 313.82	36.44 ± 27.01	13.24 ± 3.25	234.06 ± 190.03	0.42 ± 0.19	1.53 ± 0.25	0.98 ± 0.55
30 mg	550.83 ± 197.82	39.21 ± 34.21	11.87 ± 3.60	123.26 ± 138.21	0.29 ± 0.15	1.53 ± 0.21	1.59 ± 0.82
SEM*	52.57	5.67	0.609	39.08	0.034	0.040	0.137
CV	43.44	79.52	27.47	87.05	49.54	15.06	58.67

Source of variations				*p*‐value			
Vit B6	0.65	0.01	0.01	0.01	0.85	0.03	0.69
MgSO_4_	0.23	0.06	0.01	0.01	0.07	0.04	0.01
ZnO	0.13	0.19	0.10	0.02	0.06	0.97	0.01
Vit B6 × MgSO_4_	0.95	0.01	0.33	0.01	0.52	0.20	0.05
Vit B6 × ZnO	0.02	0.38	0.51	0.01	0.34	0.01	0.01
MgSO_4_ × ZnO	0.38	0.01	0.41	0.24	0.23	0.96	0.01
Three‐way interaction	0.69	0.03	0.01	0.72	0.97	0.01	0.25

Values in the same column with different superscripts are significantly different (*p* < 0.05).

* SEM, standard error of the mean.

^a^ Heat‐stress condition.

**TABLE 5:** Interaction between Vit B6 (a), Mg (b) and Zinc (c) on egg weight, Haugh unit, shell weight, shell thickness, and blood concentrations of insulin, Zn and Mg of laying hens reared under normal (weeks 30 to 40 of age) and heat‐stress (weeks 41 to 45 of age) conditions

	Egg weight (g)	Haugh unit	Shell weight (g)	Shell thickness (mm^2−^ × 10)	Insulin (μIU/mL)	Zn (μg/mL)	Mg (μg/mL)
Age (week)	45‐40	30‐40	45‐40	45‐40	45‐40	45‐40	45‐40
Condition	HSC^a^	NC^b^	HSC	HSC	HSC	HSC	HSC
a_0_b_0_c_0_	58.73 ± 1.63^a^, ^b^	67.05 ± 2.28^a‐c^	5.46 ± 0.28^a^, ^b^	0.33 ± 0.22^a^, ^b^	9.26 ± 2.03^d^	13.32 ± 0.94^a^, ^b^	1.72 ± 0.02^a^
a_0_b_0_c_1_	58.48 ± 0.90^b^	69.25 ± 2.96^b^, ^a‐c^	5.55 ± 0.17^a^	0.34 ± 0.008^a^	20.75 ± 3.25^d^ ^a‐c^	15.96 ± 2.25^a^	1.72 ± 0.11^a^
a_0_b_1_c_0_	57.77 ± 1.40^b^	71.38 ± 1.64^a^, ^b^	5.54 ± 0.25^a^	0.35 ± 0.16^a^	39.96 ± 25.11^b^, ^a‐c^ ^d^	15.92 ± 2.33^a^	1.65 ± 0.12^a^
a_0_b_1_c_1_	58.93 ± 1.36^a^, ^b^	70.63 ± 4.02^a^, ^b^	5.41 ± 0.34^a^, ^b^	0.33 ± 0.010^a^, ^b^	34.95 ± 17.31^b^, ^a‐c^ ^d^	9.50 ± 3.44^a‐c^	1.45 ± 0.26^b^, ^a‐c^
a_1_b_0_c_0_	58.17 ± 1.90^b^	72.68 ± 3.62^a^	5.58 ± 0.31^a^	0.35 ± 0.008^a^	55.24 ± 11.10^b^	15.20 ± 1.37^a^	1.48 ± 0.24^a^, ^b^, ^a‐c^
a_1_b_0_c_1_	58.91 ± 0.65^a^, ^b^	70.94 ± 2.98^a^, ^b^	5.13 ± 0.37^b^	0.32 ± 0.024^a^, ^b^	112.08 ± 4.36^a^	11.17 ± 1.96^b^, ^a‐c^	1.48 ± 0.15^a^, ^b^, ^a‐c^
a_1_b_1_c_0_	60.03 ± 1.21^a^	68.89 ± 1.66^b^, ^a‐c^	5.41 ± 0.19^a^, ^b^	0.33 ± 0.008^b^	46.02 ± 34.78^b^, ^a‐c^	8.50 ± 0.68^a‐c^	1.26 ± 0.28^a‐c^
a_1_b_1_c_1_	58.53 ± 1.60^a^, ^b^	70.98 ± 3.08^a^, ^b^	5.49 ± 0.34^a^	0.34 ± 0.013^a^	20.91 ± 4.18^a‐c^ ^d^	10.86 ± 3.52^a‐c^	1.60 ± 0.08^a^, ^b^

^a‐c^ Mean values in the same column having different superscripts are significantly different (*p* < 0.05).

^a^ Heat‐stress condition.

^b^ Normal condition.

The egg shape index was increased by Zn as a single supplement before heat‐stress exposure (*p* < 0.05), and tended to be increased during heat‐stress exposure (*p* = 0.06) but there were no effects for other treatments. Salim et al. (2012b) showed that diet supplementation with organic Zn increased the plasma concentration of Ca in broiler chickens. In addition, before heat‐stress exposure, it was demonstrated that yolk index was decreased by single supplementation of Zn. Interaction effects between the Vit B6 (a), Mg (b) and Zinc (c) on yolk index values were significant in normal (weeks 30 to 40 of age) and heat‐stress (weeks 41 to 45 of age) conditions (Table 6). Carbonic anhydrase enzyme supplies the carbonate ions during eggshell formation (Nys et al., 2001); the definite role of Zn in this enzyme may be the reason for egg shape index improvement in Zn‐supplemented laying hens before heat stress. This research indicated that the yolk color was decreased through feeding Mg before heat‐stress exposure. In several countries, egg yolk color is an important factor for egg marketing because many consumers prefer a golden yellow to pale yellow color. Egg yolk pigmentation is a practical subject for the egg production industry, which requires the production of eggs with an appropriate pigmentation level and homogeneous distribution of color to satisfy food industry demands (Sachchidananda et al., 2008). Because laying hens cannot synthesize egg yolk pigments, egg yolk color closely depends on the fat‐soluble pigments in the diets fed (Rose, 2005). In addition, *β*‐carotene in poultry is almost completely converted to vitamin A or is otherwise metabolized, oxycarotenoids (xanthophylls) in feed ingredients play a major role in egg yolk pigmentation (Nui et al., 2008). The primary sources of xanthophylls are corn, corn gluten meal and dehydrated alfalfa meal (Bailey & Chen, 1989). In this experiment, it can be assumed that reduced FI (even numerically), which causes reduced pigment intake by the birds, leads to significant reduction in yolk color. Definitely, several variables, such as supplementation methods (in diet or drinking water), the supplemental level, its level in the basal diet, bioavailability, stress condition, degree of stress, as well as duration of usage could be, at least in part, probable reasons for various results in miscellaneous experiments.

**TABLE 6:** Interaction between Vit B6 (a), Mg (b) and Zinc (c) on yolk index, and blood concentrations of triglycerides of laying hens reared under normal (weeks 30 to 40 of age) and heat‐stress (weeks 41 to 45 of age) conditions

	Yolk index (%)	Yolk index (%)		Triglycerides (mg/dL)		Triglycerides (mg/dL)		Triglycerides (mg/dL)		Triglycerides (mg/dL)
Age (week)	30‐40	40–45		30–40		30–40		30—40		40–45
Condition	NC^a^	HSC^b^		NC		NC		NC		HSC
b_0_c_0_	41.88 ± 1.73^a^	37.23 ± 1.52^a^, ^b^	a_0_b_0_	1614.65 ± 417.22^b^	a_0_c_0_	1606.99 ± 380.23^b^	b_0_c_0_	1711.93 ± 558.79^b^	a_0_c_0_	1497.50 ± 433.36^a‐c^
b_1_c_0_	39.99 ± 1.49^b^	37.78 ± 0.95^a^, ^b^	a_1_b_0_	1987.54 ± 252.62^a^	a_1_c_0_	2045.05 ± 239.12^a^	b_1_c_0_	2160.41 ± 204.31^a^	a_1_c_0_	2077.37 ± 433.80 ^b^
b_0_c_1_	41.10 ± 1.03^a^, ^b^	38.08 ± 0.93^a^	a_0_b_1_	2212.54 ± 349.56^a^	a_0_c_1_	2007.39 ± 371.38^b^	b_0_c_1_	1891.96 ± 161.45^b^	a_0_c_1_	2531.65 ± 390.96^a^
b_1_c_1_	41.04 ± 1.81^a^, ^b^	37.12 ± 0.87^b^	a_1_b_1_	1586.68 ± 283.26^b^	a_1_c_1_	1791.84 ± 506.26^b^	b_1_c_1_	16.48.35 ± 429.84^b^	a_1_c_1_	1726.47 ± 598.19^b^, ^a‐c^

^a‐c^ Mean values in the same column having different superscripts are significantly different (*p* < 0.05).

^a^ Normal condition.

^b^ Heat‐stress condition.

### 3.3 Blood parameters

The effects of the supplements on TG, cholesterol and total protein concentrations in serum of the laying hens are presented in Table 7. The serum TG concentration was decreased when Mg was used as a single supplement after heat‐stress exposure. In this study, feeding Zn did not influence serum TG concentration either before or after heat‐stress exposure. Interaction effects between Vit B6 (a), Mg (b) and Zinc (c) on triglycerides levels were significant in normal (weeks 30 to 40 of age) and heat‐stress (weeks 41 to 45 of age) conditions (Table 6). Interaction effects between the Vit B6 (a), Mg (b) and Zinc (c) on Fe and Cu levels were significant in heat‐stress (weeks 41 to 45 of age) conditions (Table 8). Interaction effects between Vit B6 (a), Mg (b) and Zinc (c) on Fe and Cu levels were significant in heat‐stress (weeks 41 to 45 of age) condition (Table 8). Kucuk (2008) showed that the addition of 30 mg/kg Zn (as zinc acetate) lowered serum TG concentration in heat‐stressed broiler chickens, which is in contrast with our research. The Zn supplement in our study was zinc oxide, but in Kucuk's study, it was zinc acetate. We can assume that various forms of supplements are, at least in part, probable reasons for different results in different experiments. In this experiment, feeding Vit B6 decreased cholesterol level before heat stress, but increased TG level after heat stress. In agreement, in a study, feeding Vit B6 (16 ppm) with corn oil (3%) decreased cholesterol level and increased TG (Kucuk, 2012). In contrast, found that the levels of free and total plasma cholesterol increased with the daily intake of Vit B6 (0.05, 0.10, 0.50, 1 and 2 mg) for 2 months in rhesus monkeys. Moreover, Adekunle & Adedeji (2011) showed that there was a significant decrease in the level of cholesterol in rats that were administered with Vit B6 as compared to the control animals. Vitamin B6 is involved in lipid metabolism (Kucuk, 2012). It is shown that deficiency of Vit B6 may impair the metabolism of (n‐3) PUFA from alpha linolenic acid to eicosapentaenoic acid and docosahexaenoic acid (DHA), with the most pronounced reduction in the production of DHA (Tsuge et al., 2000).

**TABLE 7:** Interaction between Vit B6 (a), Mg (b) and Zinc (c) on blood concentrations of triglycerides, cholesterol, total protein and glucose of laying hens reared under normal (weeks 30 to 40 of age) and heat‐ stress (weeks 41 to 45 of age) conditions

	Triglycerides (mg/dL)		Cholesterol (mg/dL)		Total protein (g/dL)		Glucose (mg/dL)		Glucose (mg/dL)		Glucose (mg/dL)
Age (week)	40–45		30—40		30–40		30–40		30–40		40–45
Condition	HSC^a^		NC^b^		NC		NC		NC		HSC
b_0_c_0_	1556.77 ± 645.19^b^	b_0_c_0_	196.82 ± 31.54^b^	a_0_c_0_	4.78 ± 0.65^a‐c^	b_0_c_0_	517.44 ± 135.89^a^	b_0_c_0_	405.11 ± 232.06^b^	a_0_c_0_	419.22 ± 183.17^a^
b_1_c_0_	2045 ± 669.27^a^, ^b^	b_1_c_0_	312.15 ± 100.02^a^	a_1_c_0_	6.18 ± 0.86^a^	b_1_c_0_	384.85 ± 86.94^b^	b_1_c_0_	661.26 ± 157.47^a^	a_1_c_0_	319.25 ± 71.53^b^
b_0_c_1_	2357.47 ± 462.43^a^	b_0_c_1_	264.90 ± 80.35^a^	a_0_c_1_	5.53 ± 0.86^a^, ^b^	b_0_c_1_	246.81 ± 55.55^a‐c^	b_0_c_1_	474.50 ± 115.51^b^	a_0_c_1_	295.80 ± 125.11^b^
b_1_c_1_	1779.55 ± 332.81^b^	b_1_c_1_	208.36 ± 44.34^b^	a_1_c_1_	5.24 ± 0.70^b^ ^c^	b_1_c_1_	281.52 ± 47.89^a‐c^	b_1_c_1_	527.07 ± 188.47^a^, ^b^	a_1_c_1_	310.11 ± 86.84^b^

^a‐c^ Mean values in the same column having different superscripts are significantly different (*p* < 0.05).

^a^ Heat‐stress condition.

^b^ Normal condition.

**TABLE 8:** Interaction between Vit B6 (a), Mg (b) and Zinc (c) on plasma glutathione peroxidase activity, and Fe and Cu of laying hens reared under heat‐stress (weeks 41 to 45 of age) condition

	Glutathione peroxidase activity		Fe (μg/mL)	Cu (μg/mL)		Fe (μg/mL)	Cu (μg/mL)		Cu (μg/mL)
Age (week)	40–45		40–45	40–45		40–45	40–45		40–45
Condition	HSC^a^		HSC	HSC		HSC	HSC		HSC
a_0_c_0_	609.51 ± 302.71^a^	a_0_b_0_	409.80 ± 118.69^a^	0.90 ± 0.41^a‐c^	a_0_c_0_	361.35 ± 141.32^a^	0.77 ± 0.19^a‐c^	b_0_c_0_	1.10 ± 0.76^a‐c^
a_1_c_0_	692.58 ± 162.98^a^	a_1_b_0_	151.87 ± 136.48^a‐c^	1.66 ± 0.98^a^	a_1_c_0_	170.40 ± 176.71^a‐c^	1.80 ± 0.91^a^	b_1_c_0_	0.99 ± 0.38^a‐c^
a_0_c_1_	784.71 ± 322.16^a^	a_0_b_1_	69.60 ± 54.13^a‐c^	1.23 ± 0.74^a‐c^	a_0_c_1_	53.82 ± 25.24^a‐c^	1.20 ± 0.71^a‐c^	b_0_c_1_	0.87 ± 0.21^a‐c^
a_1_c_1_	409.07 ± 106.30b	a_1_b_1_	57.20 ± 81.18^a‐c^	1.29 ± 0.67^a^, ^a‐c^	a_1_c_1_	68.28 ± 40.46^a‐c^	1.35 ± 0.68^a‐c^	b_1_c_1_	2.29 ± 0.60^a^

^a‐c^ Mean values in the same column having different superscripts are significantly different (*p* < 0.05).

^a^ Heat‐stress condition.

Trace elements are important for the proper functioning of a number of enzymes and proteins that are involved in many physiological, biochemical and metabolic processes that contribute to the growth and production. Overall, trace elements improve immune competence and productivity (Yatoo et al., 2013). Zinc is an essential trace element that plays an important role in the regulation of protein and nitrogen metabolism as well as in anti‐oxidant defense. The primary roles of Zn are cell replication and amino acid metabolism (Szabo et al., 1999). While administration of zinc oxide with Vit B6 leads to a significant increase in total protein and albumin which could be attributed to the anti‐radicals, anti‐oxidant and metal‐chelating efficacy of this element (Derouiche & Kechrid, 2013). In addition, these findings agreed with those obtained by other studies that postulated the beneficial roles of Zn on histological and enzymatic changes in rats (Djemli et al., 2012). These reports emphasized the hepato‐protective efficacy of Zn, as Zn treatment helps in the maintaining the homeostasis through regulation of protein synthesis.

The serum total protein concentration was increased when Zn was used as single dietary supplement before heat‐stress exposure (*p* < 0.05), but it tended to decrease during heat‐stress exposure (*p* = 0.07). In addition, no significant effect of dietary Vit B6 and Mg alone was detected on serum total protein concentration either before or during heat‐stress exposure. In other studies, Salim et al. (2012a) reported that the serum total protein concentration was not affected by the supplementation of 25 mg/kg Zn from an organic source, whereas Feng et al. (2010) showed that serum total protein concentration of broilers was increased by dietary addition of 30 mg/kg Zn either from zinc‐glycine or zinc‐sulfate. Also, Kucuk et al. (2003) reported that serum total protein concentration of heat‐stressed broiler chickens was increased by supplementation of 30 mg/kg Zn (as zinc sulfate) to diet. Similarly, Sahin et al. (2002) showed that serum total protein concentration of laying hens reared at a low ambient temperature was increased by Zn supplementation. Under heat stress, the concentration of serum total protein and albumin can be increased due to dehydration because of increased breathing rate (Erickson et al., 2001). In this research, the level of serum albumin was not affected by the supplements before heat‐stress exposure. In agreement, Salim et al. (2012a) reported that the serum albumin concentration of broiler chickens was not affected by the addition of 25 mg/kg Zn to the diet. However, the serum albumin concentration was increased when Zn was used as a single supplement during heat‐stress exposure, but this effect was not significant. Under heat‐ stress exposure, when Vit B6 and Mg used separately as a single supplement, there was no significant increase in EW, however, using the combination of Vit B6 and Mg there was significant increase in EW. In a study by M. Skrivan et al. (2016), they found that increasing the level of dietary Mg (1.52 to 4.0 g/kg diet) increased EW significantly which is not consistent with our results. The source (MgO) and the amount of Mg should be taken into account that may be the reason for this discrepancy.

In this research, the concentration of insulin in heat‐stressed laying hens was increased by supplemental Vit B6 and Zn (*p* < 0.05). According to Takatori et al. (2004), pyridoxamine improves insulin secretion, glycemic control and beta‐cell regeneration in diabetic animals. In an experimental model of diabetes, hamsters treated with pyridoxamine for 4 weeks had fasting blood sugar and glucose tolerance test results similar to those of control animals without diabetes. Even more amazingly, insulin secretion improved and beta cells began to regenerate and grow in pyridoxamine‐treated diabetic animals (Takatori et al., 2004). In this study, supplemental Vit B6, Mg Zn reduced plasma glucose during heat stress. However, before heat‐stress exposure, only the treatment of Mg supplementation could reduce blood glucose. In a research, Vit B6 alone at 8 ppm, and low level of corn oil with low level of Vit B6 tended to decrease plasma glucose concentrations (Kelso et al., 2011). Kucuk et al. (2003) reported that the supplementation of 30 mg/kg Zn decreased serum glucose concentration of heat‐stressed broiler chickens.

Several mechanisms have been suggested to explain the association between Zn and insulin resistance. Zinc is known to play a major role in the stabilization of insulin hexamers and in the pancreatic storage of insulin because it can enhance insulin binding to hepatocyte membranes (Wijesekara et al., 2009). In fact, reduced hepatic insulin binding to hepatocyte membranes during Zn deficiency may be associated with the contribution of Zn during insulin receptor synthesis (Faure et al., 1992). Furthermore, Zn is an efficient anti‐oxidant, and oxidative stress is considered to be a primary contributor to the initiation and progression of insulin resistance and diabetes (Wiernsperger, 2003). In addition, Zn influences the ability of beta cells to produce and secrete insulin by playing a crucial role in synthesis, storage and secretion of insulin, as well as conformational integrity of insulin in the hexameric form (Salgueiro et al., 2001). Zn deficiency has been reported to result in an impairment of glucose tolerance in rats because of the known role of Zn associated with stored insulin (Linder, 1991).

In this experiment, Mg decreased glucose concentration. The decrease in plasma glucose could, therefore, be attributed to the direct effect of Mg on glucose disposal, since Mg is important for the action of the rate limiting enzymes of glycolysis (Altura, 1982). In a study, plasma insulin concentration was decreased following Mg administration to diabetic and non‐diabetic subjects. The results showed a significant increase in insulin sensitivity, even though to a lesser extent in diabetics. Interestingly, beta cells response was decreased in non‐diabetics, whereas it was not affected in diabetic subjects. The overall effect of Mg administration on plasma glucose and insulin is, therefore, the increase in glucose disposal as a result of an increase in insulin activity (Paolisso et al., 1992). In fact, hypermagnesemia inhibits insulin secretion (Zofkova et al., 1988), while it enhances insulin sensitivity through an increase in the binding affinity of insulin to its receptors (Paolisso et al., 1992).

The results of the present study showed that single supplementation of Vit B6 and Zn did not increase plasma glutathione peroxidase activity significantly. However, the serum glutathione peroxidase activity was decreased in hens when the combination of Vit B6 and Zn was given; besides interaction effects between the Vit B6 (a), Mg (b) and Zinc (c) on glutathione peroxidase activity were significant in heat‐stress (weeks 41 to 45 of age) condition (Table 8). Zago and Oteiza (2001) defined Zn as an important component of an anti‐oxidant network that prevents membrane damage from oxidation and also reported that both Zn and vitamin E partially inhibited malondialdehyde formation, but the simultaneous presence of both anti‐oxidants had a higher protective action. Zn is a co‐factor of the main anti‐oxidative enzyme Cu/Zn‐superoxide dismutase. Zn may play a key role in the suppression of free radicals and in the inhibition of NADPH‐dependent lipid peroxidation (Prasad, 1997), as well as in the prevention of lipid peroxidation via inhibiting glutathione depletion (Gibbs et al., 1985). Regeneration of vitamin E from α‐chromanoxyl radical appears to be mediated by glutathione and vitamin C (Kagan et al., 1992).

The plasma concentration of Fe was decreased by supplemental Vit B6, Mg and Zn, and the effect of each supplement was similar to the combination of them. However, the plasma concentration of copper was increased by the addition of single and combination of the aforementioned supplements to the diets, except the combination of Mg and Zn which decreased plasma concentration of copper. In addition, the plasma concentration of Mg was decreased by adding the combination of Vit B6 and Mg, as well as the combination of Mg and Zn to the diet. In a study, adding Zn as organic source significantly increased plasma Zn, it may be due to the increased availability of zinc methionine compared to zinc sulphate. While there were no significant differences in plasma Fe due to addition of different Zn sources.

## 4 CONCLUSIONS

From the above results, it is possible to conclude that dietary supplementation of pyridoxine, Mg and Zn did not improve production performance of laying hens either in NC or HSC. Vit B6 could improve egg weights and suppress blood cholesterol in NC; besides. Zn improved egg shape index in NC.

## ANIMAL WELFARE STATEMENT

All experimental protocols adhered to the guidelines, which were approved by, the Animal Ethics Committee of XXXX and were in accordance with the EU standards for the protection of animals and/or feed legislation.

## DISCLOSURE STATEMENT

No potential conflict of interest was reported by the authors.

## AUTHOR CONTRIBUTION

***Hossein Gholizadeh***: Performed experiment, collected and statistically analysed the experimental data, prepared the result tables.

***Mehran Torki***: Designed and supervised the research, wrote the article.

***Hamed Mohammadi***: Co‐wrote the article.

## STATEMENT ON ETHICAL APPROVAL

All experimental protocols adhered to the guidelines, which were approved by, the Animal Ethics Committee of the Razi University, Kermanshsh, Iran, and were in accordance with the EU standards for the protection of animals and/or feed legislation.

### PEER REVIEW

The peer review history for this article is available at https://publons.com/publon/10.1002/vms3.737

## Data Availability

The data that supports the findings of this study are available on the request of readers via the email address provided.

## References

[vms3737-bib-0001] Adekunle, A. S. , & Adedeji, A. L. (2011). Anti‐atherogenic effects of supplementation with vitamin B6 (pyridoxine) in albino rats. African Journal of Biochemistry Research, 5(13), 352–355.

[vms3737-bib-0002] Altura, B. M. (1982). Magnesium and regulation of contractility of vascular smooth muscle. Adv Mcrocirc, 11, 77–113.

[vms3737-bib-0003] Anderson, G. H. , & Johnston, J. L. (1983). Nutrient control of brain neurotransmitter synthesis and function. Canadian Journal of Physiology and Pharmacology, 61, 271–281. 6132676 10.1139/y83-042

[vms3737-bib-0004] Atteh, J. O. , & Leeson, S. (1982). Influence of increasing dietary calcium and magnesium levels on performance, mineral metabolism, and egg mineral content of laying hens. Poultry Science, 62, 1261–1268. 10.3382/ps.0621261 6622366

[vms3737-bib-0005] Azzam, M. M. , Alhotan, R. , Al‐Abdullatif, A. , Al‐Mufarrej, S. , Mabkhot, M. , Alhidary, I. A. , & Zheng, C. (2019). Threonine requirements in dietary low crude protein for laying hens under high‐temperature environmental climate. Animals (Basel), 9(9), 586. 10.3390/ani9090586 PMC6770405 31438458

[vms3737-bib-0006] Bailey, D. , & Chen, J. N. (1989). Chromatographic analyses of xanthophylls in egg yolks from laying hens fed turf bermudagrass (*Cynodon dactylon*) meal. Journal of Food Science, 54, 584–586.

[vms3737-bib-0007] Balnave, D. , & Zhang, D. (1993). Research Note: Responses of laying hens on saline drinking water to dietary supplementation with various zinc compounds. Poultry Science, 72, 603–606. 10.3382/ps.0720603 8464801

[vms3737-bib-0009] Chand, N. , Muhammad, S. , Khan, R. U. , Alhidary, I. A. , & Rahman, Z. U. (2016). Ameliorative effect of synthetic γ‐aminobutyric acid (GABA) on performance traits, antioxidant status and immune response in broiler exposed to cyclic heat stress. Environmental Science and Pollution Research International, 23, 23930–23935. 27628921 10.1007/s11356-016-7604-2

[vms3737-bib-0010] Clark, I. (1969). Metabolic interrelations of calcium, magnesium and phosphate. American Journal of Physiology, 217, 871–878. 10.1152/ajplegacy.1969.217.3.871 5807714

[vms3737-bib-0011] Derouiche, S. , & Kechrid, Z. (2013). Influence of calcium supplements on zinc status, carbohydrate metabolism and the liver activity of detoxifying glutathione enzymatic system in alloxan‐induced diabetic rats. J Exp Biol Agr Sci, 1(6), 424–429.

[vms3737-bib-0012] Djemli, S. , Kechrid, Z. , & Mohamed, R. D. (2012). Combined protective effect of zinc and vitamin C on nickel‐induced oxidative liver injury in rats. Ann Biol Res, 3(7), 3410–3418.

[vms3737-bib-0013] Eisen, E. J. , Bohren, B. B. , & Mckean, H. E. (1962). The Haugh unit as a measure of egg albumen quality. Poultry Science, 41, 1461–1468.

[vms3737-bib-0014] Erickson, H. H. , Hildreth, T. , Poole, D. C. , & Cox, J. (2001). Management of exercise induced pulmonary hemorrhage in non racing performance horses. Compendium (Yardley, PA), 23, 1090–1093

[vms3737-bib-0015] Estrada‐Pareja, M. M. , Marquez‐Giron, S. M. , & Restrepo Betancur, L. F. (2007). Effect of temperature and relative humidity on the productive behaviour and the transfer of heat in broilers. Revista Colombiana de Ciencias Pecuarias, 20(3), 288–303.

[vms3737-bib-0083] Faure, P. , Roussel, A. , & Coudray, C. (1992). Zinc and insulin sensitivity. Biological Trace Element Research 32, 305–310. 10.1007/BF02784615 1375070

[vms3737-bib-0017] Feng, J. , Ma, W. Q. , Niu, H. H. , Wu, X. M. , & Wang, Y. (2010). Effects of zinc glycine chelate on growth, hematological, and immunological characteristics in broilers. Biological Trace Element Research, 133, 203–211. 19551351 10.1007/s12011-009-8431-9

[vms3737-bib-0018] Geraert, P. A. , Padilha, J. C. F. , & Guillaumin, S. (1996). Metabolic and endocrine changes induced by chronic heat exposure chickens: biological and endocrinological variables. British Journal of Nutrition, 75, 195–204. 10.1079/bjn19960125 8785199

[vms3737-bib-0019] Gibbs, P. N. , Gore, M. G. , Jordan, P. M. (1985). Investigation of the effect of metal ions on the reactivity of thiol groups in human 5‐aminolaevulinate dehydratase. Biochemical Journal, 225, 573–580. 10.1042/bj2250573 PMC1144631 3977848

[vms3737-bib-0020] Groff, J. L. , & Gropper, S. S. (2000). Advanced nutrition and human metabolism (3rd ed.). West/Wadsworth.

[vms3737-bib-0021] Hamidi, H. , Jahanian, R. , & Pourreza, J. (2010). Effect of dietary betaine on performance, immunocompetence and gut contents osmolarity of broilers challenged with a mixed coccidial infection. Asian J Anim Vet Adv, 5, 193–201.

[vms3737-bib-0022] Heisler, L. K. , Cowley, M. A. , Kishi, T. , Tecott, L. H. , Fan, W. , Low, M. J. , Smart, J. L. , Rubinstein, M. , Tatro, J. , Zigman, J. M. , Cone, R. D. , & Elmquist, J. K. (2003). Central serotonin and melanocortin pathways regulating energy homeostasis. Ann N Y Acad Sci, 994, 169–174. 12851313 10.1111/j.1749-6632.2003.tb03177.x

[vms3737-bib-0023] Holder, D. P. , & Bradford, M. V. (1979). Relationship of specific gravity of chicken eggs to number of cracked eggs observed and percent shell. Poultry Science, 58, 250–251.

[vms3737-bib-0024] Kagan, V. E. , Shvedova, A. , Serbinova, E. , Khan, S. , Swanson, C. , Powell, R. , & Packer, L. (1992). Dihydrolipoic acid —a universal antioxidant both in the membrane and in the aqueous phase. Reduction of peroxyl, ascorbyl and chromanoxyl radicals. Biochemical Pharmacology, 44, 1637–1649. 1417985 10.1016/0006-2952(92)90482-x

[vms3737-bib-0025] Karami, M. , Torki, M. , & Mohammadi, H. (2018). Effects of dietary supplemental chromium methionine, zinc oxide, and ascorbic acid on performance, egg quality traits, and blood parameters of laying hens subjected to heat stress. J Appl Anim Res, 46(1), 1174–1184. 10.1080/09712119.2018.1481411

[vms3737-bib-0082] Kazim, S. & Kucuk, O. (2003). Zinc supplementation alleviates heat stress in laying Japanese quail. The Journal of Nutrition, 133(9):2808–2811. 10.1093/jn/133.9.2808 12949369

[vms3737-bib-0026] Kelso, B. G. , Brower, J. B. , Targovnik, J. H. , & Caplan, M. R. (2011). Pyridoxine restores endothelial cell function in high glucose. Metabolic Syndrome and Related Disorders, 9, 63–68. 21034273 10.1089/met.2010.0085

[vms3737-bib-0027] Khan, R. U. , Naz, S. , & Dhama, K. (2014). Chromium: pharmacological applications in heat stressed poultry. International Journal of Pharmaceutics, 10, 213–317.

[vms3737-bib-0028] Khan, R. U. , Naz, S. , Nikousefat, Z. , Tufarelli, V. , Javadani, M. , Rana, N. , & Laudadio, V. (2011). Effect of vitamin E in heat‐stressed poultry. World's Poultry Science Journal, 67(3), 469–478.

[vms3737-bib-0029] Kidd, M. T. , Anthony, N. B. , Johnson, Z. , & Lee, S. R. (1992). Effect of zinc‐methionine supplementation on the performance of mature broiler breeders. Appl Poult Sci, 1, 207–211.

[vms3737-bib-0030] Kucuk, O. , Kahraman, A. , Kurt, I. , Yildiz, N. , & Onmaz, A. C. (2008). A combination of zinc and pyridoxine supplementation to the diet of laying hens improves performance and egg quality. Biological Trace Element Research, 126(1), 165–175. 18719859 10.1007/s12011-008-8190-z

[vms3737-bib-0031] Kucuk, O. , Sahin, N. , & Sahin, K. (2003). Supplemental zinc and vitamin A can alleviate negative effects of heat stress in broiler chickens. Biological Trace Element Research, 94(3), 225–235. 12972690 10.1385/BTER:94:3:225

[vms3737-bib-0032] Kucuk, O. (2008). Zinc in a combination with magnesium helps reducing negative effects of heat stress in quails. Biological Trace Element Research, 123, 144–153. 18188513 10.1007/s12011-007-8083-6

[vms3737-bib-0033] Kucuk, O. (2012). Supplemental pyridoxine had no influence on the live performance and, blood parameters, except glucose, in quails fed 3 or 6% corn oil. J Health Sci, 21(1), 38–44.

[vms3737-bib-0034] Laudadio, V. , Passantino, L. , Perillo, A. , Lopresti, G. , Passantino, A. , Khan, R. U. , & Tufarelli, V. (2012). Productive performance and histological features of intestinal mucosa of broiler chickens fed different dietary protein levels. Poultry Science, 91, 16. 10.3382/ps.2011-01675 22184453

[vms3737-bib-0035] Lee, S. , & Britton, W. M. (1980). Magnesium toxicity: Effect on phosphorus utilization by broiler chicks. Poultry Science, 59, 1989–1994. 10.3382/ps.0591989 7433357

[vms3737-bib-0036] Li, L. L. , Gong, Y. J. , Zhan, H. Q. , Zheng, Y.X. , & Zou, X. T. (2019). Effects of dietary Zn‐methionine supplementation on the laying performance, egg quality, antioxidant capacity, and serum parameters of laying hens. Poult Scie, 98(2), 923–931. 10.3382/ps/pey440 30299460

[vms3737-bib-0037] Linder, M. C. (1991). Nutrition and metabolism of the trace elements. In M. C. Linder (Ed.), Nutritional biochemistry and metabolism with clinical applications (pp. 215–276). Elsevier.

[vms3737-bib-0038] Liu, Y. X. , Guo, Y. M. , Wang, Z. , & Nie, W. (2007). Effects of source and level of magnesium on catalase activity and its gene expression in livers of broiler chickens. Archives of Animal Nutrition, 61, 292–300. 17760306 10.1080/17450390701432019

[vms3737-bib-0039] Macmanus, J. , Heaton, F. W. , & Lucas, P. W. (1971). A decreased response to parathyroid hormone in magnesium deficiency. Journal of Endocrinology, 49, 253–258. 10.1677/joe.0.0490253 5091235

[vms3737-bib-0040] Mashaly, M. M. , Hendricks, G. L. , Kalama, M. A. , Gehad, A. E. , Abbas, A. O. , & Patterson, P. H. (2004). Effect of heat stress on production parameters and immune responses of commercial laying hens. Poultry Science, 83, 889–894. 10.1093/ps/83.6.889 15206614

[vms3737-bib-0041] Massry, S. R. , Coburn, S. W. , & Kleeman, C. R. (1970). Evidence for suppression of parathyroid gland activity by hypermagnesemia. Journal of Clinical Investigation, 49, 1619–1628. 10.1172/JCI106379 PMC322645 5449702

[vms3737-bib-0042] Mcdonald, P. , Edwards, R. A. , & Greenhalgh, J. F. D. (1971). Animal nutrition (3rd ed., pp. 83–84). Oliver and Boyd.

[vms3737-bib-0043] Mcdowell, L. R. (1989). *Vitamins in animal nutrition— comparative aspects to human nutrition*. Vitamin A and E (pp. 93–131). Academic Press. 10–52,

[vms3737-bib-0044] Moreng, R. E. , Balnave, D. , & Zhang, D. (1992). Dietary zinc methionine effect on eggshell quality of hens drinking saline water. Poultry Science, 71, 1163–1167. 10.3382/ps.0711163 1641380

[vms3737-bib-0045] Nachtigal, M. C. , Patterson, R. E. , Stratton, K. L. , Adams, L. A. , Shattuck, A. L. , & White, E. (2005). Dietary supplements and weight control in a middle‐age population. Journal of Alternative and Complementary Medicine, 11, 909–915. 16296926 10.1089/acm.2005.11.909

[vms3737-bib-0081] NRC: Research Council National (1994). Nutrient requirements of poultry (9th ed.). Washington, DC: The National Academies Press.

[vms3737-bib-0046] Nui, Z. , Fu, J. , Gao, Y. , & Liu, F. (2008). Influence of paprika extract supplement on egg quality of laying hens fed wheat‐based diet. Int J Poult Sci, 7, 887–889.

[vms3737-bib-0047] Nys, Y. , Gautron, J. , Garcia‐Ruiz, J. M. , & Hincke, M. (2001). Biochemical and functional characterization of eggshell matrix proteins. World's Poultry Science Journal, 57, 401–403.

[vms3737-bib-0048] Paglia, D. E. , & Valentine, W. N. (1967). Studies on the quantitative and qualitative characterization of erythrocyte glutathione peroxidase. Journal of Laboratory and Clinical Medicine, 70, 158–169. 6066618

[vms3737-bib-0049] Paolisso, G. , Giugliano, D. , Pizza, G. , Gambardella, A. , Tesauro, P. , Varricchio, M. , & D'onofrio, F. (1992). Glutathione infusion potentiates glucose‐induced insulin secretion in aged patients with impaired glucose tolerance. Diabetes Care, 15, 1–7. 10.2337/diacare.15.1.1 1737525

[vms3737-bib-0050] Paulicks, B. R. , Kirchgessner, M. (1994). Influence of supply on feed intake and performance of layers. Archiv für Geflügelkunde, 58, 186–191.

[vms3737-bib-0051] Prasad, T. K. (1997). Role of catalase in inducing chilling tolerance in preemergent maize seedlings. Plant Physiology, 114, 1369–1376. 12223775 10.1104/pp.114.4.1369 PMC158429

[vms3737-bib-0052] Puthpongsiriporn, U. S. , Scheideler, E. , Sell, J. L. , & Beck, M. M. (2001). Effects of vitamin E and C supplementation on performance, in vitro lymphocyte proliferation, and antioxidant status of laying hens during heat stress. Poultry Science, 80, 1190–1200. 10.1093/ps/80.8.1190 11495472

[vms3737-bib-0053] Rose, S. P. (2005). Principles of poultry science. CAB Int.

[vms3737-bib-0054] Sachchidananda, D. C. , Begum, M. H. , Shubash, C. D. , Rashid, M. H. , & Ferdaus, A. J. M. (2008). Evaluation of marigold flower and orange skin as sources of xanthophylls pigment for the improvement of egg yolk color. Poultry Science, 45, 265–272.

[vms3737-bib-0055] Skrivan, M. , Englmaierova, M. , Marounek, M. , Skrivanova, V. , Taubner, T. , & Vit, T. (2016). Effect of dietary magnesium, calcium, phosphorus, and limestone grain size on productive performance and eggshell quality of hens. Czech J Anim Sci, 61(10), 473–480

[vms3737-bib-0056] Sahin, K. , & Kucuk, O. (2003a). Heat stress and dietary vitamin supplementation of poultry diets. Nutr Abst Rev Ser B Livest Feeds Feed, 73, 41–50.

[vms3737-bib-0057] Sahin, K. , & Kucuk, O. (2003b). Zinc supplementation alleviates heat stress in laying Japanese quail. Journal of Nutrition, 33, 2808–2811. 10.1093/jn/133.9.2808 12949369

[vms3737-bib-0058] Sahin, N. , Onderci, M. , & Sahin, K. (2002). Effects of dietary chromium and zinc on egg production, egg quality, and some blood metabolites of laying hens reared under low ambient temperature. Biological Trace Element Research, 85, 47–58. 11881798 10.1385/BTER:85:1:47

[vms3737-bib-0059] Salgueiro, M. J. , Krebs, N. , Zubillaga, M. B. , Weill, R. , Postaire, E. , Lysionek, A. E. , Caro, R. A. , Paoli, T. D. , Hager, A. , & Boccio, J. (2001). Zinc and diabetes mellitus. is there a need of zinc supplementation in diabetes mellitus patients? Biological Trace Element Research, 81, 215–228. 11575679 10.1385/BTER:81:3:215

[vms3737-bib-0060] Salim, H. , Lee, H. , Jo, C. , Lee, S. , & Lee, B. (2012a). Effect of dietary zinc proteinate supplementation on growth performance and skin and meat quality of male and female broiler chicks. British Poultry Science, 53, 116–124. 10.1080/00071668.2012.658757 22404812

[vms3737-bib-0061] Salim, H. , Lee, H. , Jo, C. , Lee, S. , & Lee, B. (2012b). Effect of sex and dietary organic zinc on growth performance, carcass traits, tissue mineral content, and blood parameters of broiler chickens. Biological Trace Element Research, 147, 120–129. 22167309 10.1007/s12011-011-9282-8

[vms3737-bib-0062] Sedgh‐Gooya, Sh. , & Torki, M. (2018). Influence of dietary supplemental chromium and magnesium on performance and metabolic parameters of laying hens subjected to heat stress. J Appl Anim Res, 46(1), 1469–1477. 10.1080/09712119.2018.1535436

[vms3737-bib-0063] Shah, M. , Zaneb, H. , Masood, S. , Khan, R. U. , Ashraf, S. , Sikandar, A. , Rehman, H. F. U. , & Rehman, H. U. (2018). Effect of dietary supplementation of zinc and multi‐microbe probiotic on growth traits and alteration of intestinal architecture in broiler. Probiotics and Antimicrobial Proteins, Apr 22. 10.1007/s12602-018-9424-9 29680883

[vms3737-bib-0064] Shastak, Y. , & Rodehutscord, M. (2015). A review of the role of magnesium in poultry nutrition. World's Poultry Science Journal, 71(1), 125–138. 10.1017/S0043933915000112

[vms3737-bib-0065] Sherwood, L. M. , Herrmann, I. , & Bassett, C. A. (1970). Parathyroid hormone secretion in vitro: Regulation by calcium and magnesium ions. Nature, 225, 1056–1057. 5416477 10.1038/2251056a0

[vms3737-bib-0066] Stafford, J. E. H. , & Edwards, N. A. (1973). Magnesium metabolism in the laying fowl. British Poultry Science, 14, 137–148. 10.1080/00071667308416006 4698867

[vms3737-bib-0067] Szabo, G. , Chavan, S. , Mandrekar, P. , & Catalano, D. (1999). Acute alcoholic consumption attenuates IL‐8 and MCP‐1 induction in response to ex Vivo stimulation. Journal of Clinical Immunology, 19, 67–76. 10080106 10.1023/a:1020518703050

[vms3737-bib-0068] Takatori, A. , Ishii, Y. , Itagaki, S. , Kyuwa, S. , & Yoshikawa, Y. (2004). Amelioration of the beta‐cell dysfunction in diabetic APA hamsters by antioxidants and AGE inhibitor treatments. Diabetes Metabolism Research and Reviews, 20(3), 211–218. 15133752 10.1002/dmrr.428

[vms3737-bib-0069] Tsuge, H. , Hotta, N. , & Hayakawa, T. (2000). Effects of vitamin B‐6 on (n‐3) polyunsaturated fatty acid metabolism. Journal of Nutrition, 130, 333S‐334S. 10.1093/jn/130.2.333S 10721899

[vms3737-bib-0070] Underwood, E. J. , & Suttle, N. F. (1999). The mineral nutrition of livestock (3rd ed., p. 497). CABI Publishing.

[vms3737-bib-0071] Vuilleumier, J. P. (1969). The ‘Roche yolk colour fan’—an instrument for measuring yolk colour. Poultry Science, 48, 767–779.

[vms3737-bib-0072] Weber, G. (2009). Improvement of flock productivity through supply of vitamins for higher laying performance and better egg quality. World's Poultry Science Journal, 65(3), 443–458.

[vms3737-bib-0073] Wiernsperger, N. F. (2003). Oxidative stress as a therapeutic target in diabetes: revisiting the controversy. Diabetes & Metabolism, 29, 579–585. 14707886 10.1016/s1262-3636(07)70072-1

[vms3737-bib-0074] Wijesekara, N. , Chimienti, F. , & Wheeler, M. B. (2009). Zinc, a regulator of islet function and glucose homeostasis. Diabetes, Obesity & Metabolism, 11(Suppl 4), 202–214. 10.1111/j.1463-1326.2009.01110.x 19817803

[vms3737-bib-0075] Wolfenson, D. , Bachrach, D. , Maman, M. , Graber, Y. , & Rozenboim, I. (2001). Evaporative cooling of ventral regions of the skin in heat‐stressed laying hens. Poultry Science, 80, 958–964. 10.1093/ps/80.7.958 11469662

[vms3737-bib-0076] Yatoo, M. I. , Saxena, A. , Kumar, P. , Gugjoo, M. B. , Dimri, U. , Sharma, M. C. , & Jhambh, R. (2013). Evaluation of serum mineral status and hormone profile in goats and some of their inter‐relations. Vet World, 6(6), 318–320.

[vms3737-bib-0077] Xing, S. , Wang, X. , Diao, H. , Zhang, M. , Zhou, Y. , & Feng, J. (2019). Changes in the cecal microbiota of laying hens during heat stress is mainly associated with reduced feed intake. Poultry Science, 98(11), 5257–5264. 10.3382/ps/pez440 31399742

[vms3737-bib-0078] Zago, M. , & Oteiza, P. I. (2001). The antioxidant properties of zinc: interactions with iron and antioxidants. Free Radical Biology & Medicine. 31, 266–274. 11440839 10.1016/s0891-5849(01)00583-4

[vms3737-bib-0079] Zofkova, I. , Zamrazil, V. , & Simeckova, A. (1988). Acute hypermagnesaemia retards glucose assimilation and inhibits insulin secretion during intravenous glucose tolerance test (IVGTT). Hormone and Metabolic Research, 20, 120–121. 3286450 10.1055/s-2007-1010768

